# Responses to persuasive messages encouraging professional help seeking for depression: comparison between individuals with and without psychological distress

**DOI:** 10.1186/s12199-019-0786-8

**Published:** 2019-05-08

**Authors:** Machi Suka, Takashi Yamauchi, Hiroyuki Yanagisawa

**Affiliations:** Department of Public Health and Environmental Medicine, The Jikei University School of Medicine, 3-25-8 Nishi-Shimbashi, Minato-ku, Tokyo, 105-8461 Japan

**Keywords:** Depression, Help-seeking, Persuasive message, Questionnaire survey, Japan

## Abstract

**Background:**

The persuasive effect of health messages can depend on message features, audience characteristics, and target behaviors. The objective of this study was to compare the responses to persuasive messages encouraging professional help seeking for depression between individuals with and without psychological distress.

**Methods:**

A cross-sectional web-based survey was conducted on Japanese adults aged 35–45 years, who randomly received one of three persuasive messages that aimed to promote help-seeking intentions for depression. The primary message statements were as follows: (1) depression can happen to anyone, (2) depression needs treatment, and (3) depression improves with treatment. Participants rated the messages in terms of comprehensibility, persuasiveness, emotional response, and intended future use. Help-seeking intention for depression was measured using vignette methodology before and after exposure to the messages. Eligible participants who had not received medical treatment for their mental disorders were classified as either distressed (K6 score ≥ 5, *N* = 824) or non-distressed (K6 score < 5, *N* = 1133) and analyzed.

**Results:**

No significant differences in comprehensibility or persuasiveness scores were observed between the messages, but the distressed group had significantly lower scores than the non-distressed group. Negative emotional responses such as surprise, anger, fear, sadness, guilt, and anxiety were significantly stronger when reading message 2, while a positive emotional response such as happiness was significantly stronger when reading message 3. These emotional responses were more prominent in the distressed than in the non-distressed group. After reading messages 1, 2, and 3, the proportions of participants in the distressed group who reported having a positive help-seeking intention increased by 35.1%, 32.1%, and 27.7%, respectively, and by 6.4%, 17.3%, and 15.2%, respectively in the non-distressed group. Multiple logistic regression analysis among participants having no help-seeking intention before exposure to the messages showed that message 2 had a significantly greater effect of increasing help-seeking intentions in the non-distressed group.

**Conclusion:**

The exposure to persuasive messages may promote help-seeking intentions for depression. It seems likely that loss framing will work better than neutral and gain framing. Meanwhile, the responses to persuasive messages may differ to some extent between distressed and non-distressed individuals, as individuals with psychological distress are likely to be more susceptible to persuasive messages than those without.

**Trial registration:**

Not applicable; this is not a report of intervention trial.

**Electronic supplementary material:**

The online version of this article (10.1186/s12199-019-0786-8) contains supplementary material, which is available to authorized users.

## Background

Depression can have a profound impact on daily functioning and quality of life. Early detection and appropriate treatment can promote remission, prevent relapse, and reduce the burden of the disease [1]. However, many affected individuals delay in seeking professional help and fail to receive effective treatment [2, 3]. The results of numerous studies performed to examine the effectiveness of interventions designed to promote help-seeking for depression have suggested that improvements were achievable in some aspects of help-seeking, but the effect sizes were small [4]. Thus, additional studies are needed to identify more effective approaches for improving public attitudes, intentions, and behaviors.

The study and use of communication strategies to inform and influence individual and community decisions that enhance health is referred to as health communication [5]. Communicating persuasive messages may be able to make significant contributions to solve the problem of untreated depression, but their effects would depend on the message features, audience characteristics, and target behaviors [6–8]. Previous studies suggested a potential negative effect of depression help-seeking messages (i.e., boomerang effect) [9, 10]. For more effective messaging, providers need to incorporate audience perspective to message design [11, 12]. Pretesting messages with audience members is recommended for this purpose [5].

We launched a research project to develop effective health communication interventions for encouraging help-seeking in people at risk of suicide. First, we developed rating scales to measure audience perceptions regarding the effectiveness of health messages among Japanese individuals [13]. Second, we intended to develop effective public health messages that would promote help-seeking intentions for depression [14]. From a public health perspective, increasing public knowledge about depression is essential to improve help-seeking for depression. Thus, our target audience was the general public, excluding individuals who had received medical treatment for their mental disorders. Since the audience can be either distressed or non-distressed, we needed to know whether the effectiveness of depression help-seeking messages is influenced by audience’s psychological distress level. To achieve this objective, we compared the responses to persuasive messages encouraging professional help seeking for depression between individuals with and without psychological distress.

## Methods

The Step approach to Message Design and Testing (SatMDT) [15] was applied to the development of persuasive messages in this study. The SatMDT draws on well-known social psychological theories of persuasion, decision making, and attitude-behavior relations, and proposes the four-step process for devising key aspects of message content likely to enhance message persuasiveness. The step 1 (pre-existing individual characteristics) and the step 2 (message-related characteristics) relate to developing message concepts, deciding what messages to develop, and developing the messages. The step 3 (individual responses) and the step 4 (message outcomes) relate to pretesting the messages and assessing their effectiveness. Through the four-step process, users can complete the actions to be undertaken when they develop concepts, messages, and materials [5].

The study protocol was approved by the ethics committees of the Jikei University School of Medicine (reference number 28-223(8466)) and has been conducted in accordance with the Ethical Guidelines for Medical and Health Research Involving Human Subjects by the Japanese Government.

### Messages

In accordance with the step 1 (pre-existing individual characteristics) and the step 2 (message-related characteristics), we developed three prototypes of persuasive messages. The aim of messaging was to promote help-seeking intentions for depression by imparting knowledge about depression. The target audience was the general public, excluding individuals who had received medical treatment for their mental disorders. As a general rule in Japan, people who report depressive symptoms such as depressed mood and disturbed sleep for over 2 weeks often screen positive for suspected depression, who are encouraged to seek advice and assistance from health professionals. The persuasive messages were designed to incorporate this approach.

The persuasive messages used in this study were shown in Additional file 1. Each message consisted of three parts. The first part was the primary message statement. The second part provided information regarding the early signs of depression, that is “Depression can be recognized early by mental symptoms such as depressed mood, loss of interest, etc. and physical symptoms such as disturbed sleep, increased fatigue, etc.”. The last part was a call to action, that is “if you think you might be depressed, don’t worry alone and speak with your familiar primary care doctor.”.

The three primary message statements were selected from the list of text messages devised by Bell and colleagues [16] so as to be matched with the beliefs related to the top three following reasons, respectively, for having no help-seeking intentions for depression, respectively [17]: (1) depression can happen to anyone, (2) depression needs treatment, and (3) depression improves with treatment. The first one was neutral framed with additional information on incidence of depression: about one out of 15 people experience depression during their lifetime. The second one was loss framed (thereat appeal) with additional information on prognosis of untreated patients: about 80% of untreated patients will not recover. The third one was gain framed (benefit appeal) with additional information on prognosis of treated patients: about 80% of treated patients will recover.

Formatted and unformatted versions of the three differently framed messages containing the identical sentences were prepared. The formatted versions were designed in accordance with the CDC Clear Communication Index User Guide [18], while the unformatted versions were designed in plain text. No significant differences in participants’ assessment (comprehensibility and persuasiveness) were observed between the formatted and unformatted versions [14]. Therefore, the responses were lumped together in this study.

### Participants

In accordance with the step 3 (individual responses) and the step 4 (message outcomes), we pretested the messages and assessed their effectiveness. A web-based survey was conducted in July 2017 on Japanese adults aged 35–45 years [14]. All participants were recruited from an online research panel of a leading research company in Japan (Cross Marketing Inc., Tokyo, Japan). Recruitment emails were sent to 8241 randomly selected eligible registrants, with medical professionals being excluded through a prescreening process. Potential participants in the present survey were accepted in the order of receipt until the quotas were met for gender, area, and psychological distress level as measured by the Japanese version of the 6-item Kessler Psychological Distress Scale (K6) [19]. A validation study revealed that the optimal cut-off point for the Japanese version of the K6 was estimated at 4/5 with sensitivity of 100% and specificity of 68.7% [20]. The participants were classified as either distressed (K6 score ≥ 5) or non-distressed (K6 score < 5) according to their K6 scores.

A total of 2520 responses were received over 2 days of recruitment. All participants agreed to participate in the survey voluntarily after reading a description of the purpose and procedures. Completion and submission of the survey was taken to indicate consent to participate. Of the 2520 respondents, 563 had received medical treatment for their mental disorders and therefore excluded. The remaining 1957 participants were grouped into the distressed (K6 score ≥ 5, *N* = 824) and non-distressed (K6 score < 5, *N* = 1133) groups and analyzed.

### Measures

The participants randomly received one of three persuasive messages that aimed to promote help-seeking intentions for depression, and after reading the message for at least 15 s, asked to rate it in terms of comprehensibility, persuasiveness, emotional response, and intended future use. Help-seeking intention for depression was measured using vignette methodology before and after exposure to the messages. The online questionnaire forms presented the questions one after one through the operation of a “Next” button. Respondents answered one question per page and were unable to go back to the previous page.

#### Comprehensibility

Five items on the perceived effectiveness rating scales [13] were used to ask how easy or difficult the information was to (1) read, (2) understand, (3) remember, (4) locate important information, and (5) keep for future reference. The scores for all items (range 1–5 points) were averaged to produce the comprehensibility score.

#### Persuasiveness

Seven items on the perceived effectiveness rating scales [13] were used to ask to what extent they agreed or disagreed that the information (1) was believable, (2) was convincing, (3) was important to me, (4) helped me feel confident about how best to do, (5) would help my family and friends, (6) put thoughts in my mind about wanting to do something, and (7) was agreeable. The scores for all items (range 1–5 points) were averaged to produce the persuasiveness score.

#### Emotional response

Participants were asked to what extent the message made them feel (1) surprised, (2) angry, (3) fearful, (4) sad, (5) guilty, (6) anxious, and (7) happy. The response options were from 1 (not at all) to 5 (extremely) [21, 22].

#### Intended future use

Future use of the message was measured by asking participants, “If you saw the information in a newspaper or magazine, how likely would you [use, read, and keep] it?” [17]. Participants responded to this question on a five-point scale ranging from 1 (very unlikely) to 5 (very likely).

#### Help-seeking intention for depression

Vignette methodology was used to measure help-seeking intention [13, 14, 17]. Participants were presented with a vignette describing a man (or woman) with depression and then asked “If you had health problems right now like Mr. A (or Ms. A), would you seek professional help?”. The vignette was devised to represent two core symptoms (depressed mood and loss of interest) and one associated symptom (insomnia) of major depressive disorders based on the fifth edition of the Diagnostic and Statistical Manual of Mental Disorders (DSM). A description of the vignette is as follows.

- Mr. A (Ms. A) is a 45-year-old office worker. Over the last 2 weeks, despite not having any trouble, he (she) has always been so depressed that he (she) has not felt like doing anything. He (She) has woken up frequently during the night and has not had enough sleep. Accordingly, he (she) cannot go about his (her) work. He (She) is not currently undergoing treatment for any diseases.

Participants responded to this question on a four-point scale (certainly yes/probably yes/probably not/certainly not). Those who provided affirmative answers (certainly yes and probably yes) were considered to have a positive help-seeking intention.

### Statistical analysis

All statistical analyses were performed using SAS version 9.4 (SAS Institute, Cary, NC, USA). The *t* test and χ_2_ test were used to compare characteristics of the distressed and non-distressed groups. The analysis of covariance with adjustment for sociodemographic characteristics (gender, education, marital status, and occupation) were used to assess the main and interaction effects of message and psychological distress. Proportion of variance explained (η^2^) was used to estimate the effect sizes; this is usually classified as small (0.01), medium (0.06), or large (0.14). The McNemar test was used to compare the proportions of participants who had reported having a positive help-seeking intention before and after exposure to the messages. Among participants having no help-seeking intention before exposure to the messages, multiple logistic regression analysis was performed to examine the factors associated with increased help-seeking intention for depression with adjustment for sociodemographic characteristics (gender, education, marital status, and occupation). Adjusted odds ratios (ORs) with 95% confidence intervals (CIs) for help-seeking intention for depression were calculated separately in the depressed and non-depressed groups. Significant levels were set at *p* < 0.05. In the assessment of main and interaction effects of message and psychological distress, Bonferroni adjustment was conducted for each test separately to consider the number of significance tests undertaken.

## Results

Of the 824 participants of the distressed group, those who asked to rate messages 1, 2, and 3 were 270 (32.8%), 267 (32.4%), and 287 (34.8%), respectively. Of the 1133 participants of the non-distressed group, these numbers were 382 (33.7%), 382 (33.7%), and 369 (32.6%), respectively. Table 1 shows the characteristics of the study participants. No significant differences in sociodemographic characteristics were observed between the message groups in the distressed or non-distressed groups. Despite the random allocation, significant differences were observed between the distressed and non-distressed groups in most sociodemographic characteristics, as the distressed group was significantly more likely to be younger, have lower levels of educational attainment, be unmarried, have no occupation, and have lower income than the non-distressed group.

**Table 1:** Characteristics of the study participants

		Non-distressed		Distressed		*p*
*N*		1133		824		
k6 score	Mean (SD)	1.1 (1.4)		10.3 (4.4)		< 0.001
Age	Mean (SD)	41.0 (3.0)		40.7 (3.0)		0.012
Gender	Male	570	50.3%	410	49.8%	0.810
	Female	563	49.7%	414	50.2%	
Education	Compulsory education/high school	288	25.4%	252	30.6%	0.032
	Junior college/vocational school	306	27.0%	218	26.5%	
	University or higher	539	47.6%	354	43.0%	
Marital status	Married	698	61.6%	403	48.9%	< 0.001
	Unmarried	381	33.6%	386	46.8%	
	Divorced/widowed	54	4.8%	35	4.2%	
Occupation	Full-time job	707	62.4%	469	56.9%	0.049
	Temporary or part-time job	181	16.0%	148	18.0%	
	No occupation	245	21.6%	207	25.1%	
Household income	< 2.0 million yen^a^	101	8.9%	129	15.7%	< 0.001
	2.0–3.9 million	196	17.3%	198	24.0%	
	4.0–5.9 million	333	29.4%	219	26.6%	
	6.0–7.9 million	246	21.7%	154	18.7%	
	8.0–9.9 million	140	12.4%	65	7.9%	
	10.0+ million	108	9.5%	53	6.4%	

^a^1 million yen was about 10,000 US dollars at the time of the survey

Table 2 shows the assessment of the persuasive messages. No significant differences (*p* < 0.05) in the comprehensibility and persuasiveness scores were observed between the messages, but the distressed group had significantly lower scores than the non-distressed group. Among the seven emotional responses, significant differences (*p* < 0.007) between the messages were found in fear, happiness, sadness, and anxiety, and significant differences between the distressed and non-distressed groups were found in all items except surprise. The highest scores for negative emotion items such as surprise, anger, fear, sadness, guilt, and anxiety were observed in message 2, while the highest score for a positive emotion item such as happiness was observed in message 3. Among the three items for intended future use, no significant differences (*p* < 0.016) were observed between the message, but the distressed group had a significantly higher score than the non-distressed group for keeping the message. The effect size estimates (η^2^) indicated that the effects were statistically significant but small. There were no significant interaction effects between message and psychological distress.

**Table 2:** Assessment of the persuasive messages encouraging help-seeking for depression

		Message 1		Message 2		Message 3			Effects		
		Non-distressed	Distressed	Non-distressed	Distressed	Non-distressed	Distressed		Message (A)	Distress (B)	A × B
*N*		382	270	382	267	369	287				
Comprehensibility											
Mean		3.86	3.65	3.92	3.62	3.98	3.63	η^2^	0.00	0.03	0.00
SD		0.79	0.76	0.83	0.85	0.77	0.81	p	0.497	< 0.001	0.343
Persuasiveness											
Mean		3.18	3.09	3.22	3.15	3.17	3.09	η^2^	0.00	0.00	0.00
SD		0.60	0.62	0.64	0.64	0.64	0.67	p	0.264	0.019	0.990
Emotional response											
1) Surprise	Mean	2.51	2.55	2.68	2.73	2.50	2.64	η^2^	0.00	0.00	0.00
	SD	1.08	1.04	1.08	1.00	1.10	1.00	p	0.010	0.048	0.717
2) Anger	Mean	1.85	1.99	1.88	2.12	1.78	2.16	η^2^	0.00	0.02	0.00
	SD	0.94	0.93	0.91	0.97	0.87	0.95	p	0.322	< 0.001	0.095
3) Fear	Mean	2.43	2.53	2.45	2.80	2.17	2.51	η^2^	0.01	0.02	0.00
	SD	1.03	1.05	1.07	1.02	1.00	0.97	p	< 0.001	< 0.001	0.049
4) Happiness	Mean	1.79	2.01	1.92	2.08	2.15	2.39	η^2^	0.03	0.01	0.00
	SD	0.91	0.97	0.95	1.02	0.96	0.95	p	< 0.001	< 0.001	0.736
5) Sadness	Mean	2.37	2.53	2.48	2.70	2.17	2.48	η^2^	0.01	0.01	0.00
	SD	1.08	1.03	1.04	1.04	0.94	1.00	p	< 0.001	< 0.001	0.483
6) Guilt	Mean	1.96	2.25	2.00	2.31	1.91	2.24	η^2^	0.00	0.03	0.00
	SD	0.92	0.93	0.91	0.94	0.87	0.89	p	0.266	< 0.001	0.955
7) anxiety	Mean	2.44	2.75	2.47	2.94	2.19	2.66	η^2^	0.01	0.04	0.00
	SD	1.07	1.07	1.07	1.03	1.00	0.99	p	< 0.001	< 0.001	0.308
Intended future use											
1) Read	Mean	3.23	3.16	3.36	3.16	3.22	3.27	η^2^	0.00	0.00	0.00
	SD	0.91	0.89	0.92	0.94	0.96	0.86	p	0.448	0.315	0.040
2) Use	Mean	2.82	2.63	2.85	2.85	2.78	2.82	η^2^	0.00	0.00	0.00
	SD	0.85	0.83	0.87	0.82	0.84	0.76	p	0.048	0.607	0.035
3) Keep	Mean	2.32	2.39	2.35	2.53	2.29	2.54	η^2^	0.00	0.01	0.00
	SD	0.97	0.95	0.99	0.95	0.88	0.91	p	0.289	< 0.001	0.275

All items were scored on a 1 to 5 point scale. The effects of message (A), distress (B), and A × B were estimated with adjustment for gender, education, marital status, and occupation. Effect sizes were estimated by proportion of variance explained (η^2^), which is usually classified as small 0.01, medium 0.06, and large 0.14

Table 3 shows the changes in help-seeking intention for depression before and after exposure to the messages. The proportion of participants who reported having a positive help-seeking intention for depression increased by 31.3% on average (changed from 30.2 to 39.7%) in the distressed group and by 13.0% on average (changed from 42.1 to 47.6%) in the non-distressed group. Significant increases in help-seeking intentions for depression were observed in all message groups both in the distressed and non-distressed groups.

**Table 3:** Changes in help-seeking intention for depression before and after exposure to the messages

	Non-distressed				*p*	Distressed				*p*
	*N*	Positive intention				*N*	Positive intention			
		Before	After	Percentage change			Before	After	Percentage change	
Message 1	382	157	167			270	74	100		
		41.1%	43.7%	+ 6.4%	0.258		27.4%	37.0%	+ 35.1%	< 0.001
Message 2	382	162	190			267	81	107		
		42.4%	49.7%	+ 17.3%	0.004		30.3%	40.1%	+ 32.1%	< 0.001
Message 3	369	158	182			287	94	120		
		42.8%	49.3%	+ 15.2%	0.008		32.8%	41.8%	+ 27.7%	< 0.001

Percentage change was the difference between percentages before and after exposure divided by the percentage before exposure

In order to examine the factors associated with increased help-seeking intention for depression, multiple logistic regression analysis was conducted among 1231 participants (575 distressed and 656 non-distressed) having no help-seeking intention before exposure to the messages. When the three persuasive messages were compared (Table 4), message 2 had a significantly greater effect of increasing help-seeking intentions for depression in the non-distressed group. Meanwhile, no significant differences were observed between the messages in the distressed group. When the association between message assessment and increased help-seeking intention for depression was examined (Table 5), the persuasiveness score was significantly associated with increased help-seeking intention both in the distressed and non-distressed groups. In addition, the emotional responses of “surprise” and “guilt” had a significant association with increased help-seeking intention in the non-distressed and distressed groups, respectively.

**Table 4:** Effects of increasing help-seeking intentions for depression among participants having no help-seeking intention before exposure to the messages

	Non-distressed		Distressed	
	OR	95%CI	OR	95%CI
Message 1	1.00	(reference)	1.00	(reference)
Message 2	1.67	(1.07–2.62)	0.97	(0.58–1.60)
Message 3	1.48	(0.93–2.34)	0.90	(0.54–1.49)

Odds ratios (ORs) with 95% confidence intervals (CIs) were calculated with adjustment for gender, education, marital status, and occupation

**Table 5:** Association between message assessment and increased help-seeking intention for depression among participants having no help-seeking intention before exposure to the messages

	Non-distressed		Distressed	
	OR	95% CI	OR	95% CI
Comprehensibility	1.16	(0.88–1.51)	1.38	(0.99–1.91)
Persuasiveness	1.59	(1.10–2.30)	1.77	(1.17–2.68)
Emotional responses				
1) Surprise	1.27	(1.04–1.55)	1.07	(0.83–1.37)
2) Anger	0.91	(0.66–1.23)	0.87	(0.60–1.26)
3) Fear	1.13	(0.84–1.52)	0.99	(0.70–1.39)
4) Happiness	0.84	(0.65–1.08)	1.23	(0.94–1.61)
5) Sadness	1.10	(0.83–1.46)	0.75	(0.52–1.08)
6) Guilt	1.30	(0.91–1.85)	1.62	(1.04–2.51)
7) Anxiety	0.79	(0.60–1.05)	0.84	(0.61–1.15)

Odds ratios (ORs) with 95% confidence intervals (CIs) were calculated with adjustment for gender, education, marital status, and occupation

## Discussion

This study attempted to develop effective persuasive messages in accordance with the SatMDT to promote help-seeking intentions for depression. The study participants grouped into the distressed and non-distressed groups showed significant differences in the reactions to the messages (Table 2). Meanwhile, significant increases in help-seeking intentions for depression were observed after exposure to the messages both in the distressed and non-distressed groups (Table 3). Although this study did not provide enough evidence to deny the boomerang effect of depression help-seeking messages [9, 10], it should be noted that the exposure to persuasive messages produced good results to promote help-seeking intentions for depression irrespective of whether the audience were distressed or not.

The distressed group was significantly more likely to have lower levels of educational attainment, have no occupation, and have lower income than the non-distressed group (Table 1). Although this study did not clarify their causal relationships due to the cross-sectional design, the inverse association between socioeconomic status and mental health has been observed in many countries including Japan [23, 24]. We previously reported that people with low socioeconomic status were more likely to have poor health literacy skills, less likely to get sufficient health information, and in turn, more likely to have risky health habits and poor health status [25]. It may be important to design persuasive messages targeting people with depression to be easily understandable for people with poor health literacy skills. Pretesting messages with audience members is also useful for checking the understandability of the wording of the messages.

The distressed group rated the persuasiveness of messages significantly lower than non-distressed group. Meanwhile, the emotional responses to the messages were more prominent in the distressed group, and the distressed group showed greater increases in their help-seeking intentions for depression after exposure to the messages than the non-distressed group. People with mental illness tend to have a negative attitude and low intention to seek professional help [9, 26, 27], as indeed the proportion of participants who reported having a positive help-seeking intention for depression before exposure to the messages was significantly lower in the distressed group (30.2%) than in the non-distressed group (42.1%). However, the results of this study suggested that individuals with depression may be more susceptible to the persuasive messages encouraging professional help seeking for depression. Most individuals with depression may be afraid that they need to seek medical treatment for their present depressive status, and thus they are more likely to receive the messages as self-relevant information. It seems reasonable that individuals with depression feel strongly sad when they are informed the loss-framed message, while they feel strongly happy when they are informed the gain-framed message, and also that individuals with depression think to keep the message. Recently, some investigators focused on whether increased levels of depressive symptomatology were associated with reduced help-seeking intentions [28, 29]. Since the study participants consisted of individuals with no or mild depressive symptoms, further studies may be needed to confirm the susceptibility to the messages for individuals with severe depressive symptoms.

The multiple logistic regression analysis showed that message 2 (loss-framed; negative, threat appeal) had a significantly greater effect of increasing help-seeking intentions for depression in the non-distressed group. In the World Mental Health Japan Survey, the most frequently reported reason for not seeking treatment for their mental illness was a low perceived need (63.9%) and that for delaying access to help for their mental illness was a desire to handle the problem on one’s own (68.8%) [30]. Message 2 seems to attack the major barrier of help-seeking depression directly as it was designed to emphasize the necessity of treatment. Even though no significant differences were observed between the messages in the distressed group, message 2 induced a comparative increase (32.1%) in help-seeking intentions for depression. Therefore, compared with messages 1 and 3, message 2 may be suitable for public health interventions targeting all people irrespective of whether they were depressed or not distressed. This type of public health intervention would also be likely to alter public attitudes regarding depression, and disrupt the public stigma-induced negative feedback, thereby facilitating help-seeking [31].

The results of the present study provide evidence regarding the effectiveness of depression help-seeking messages. However, the study did have potential limitations. First, the web-based survey was self-administered, so the accuracy of the responses depends on the participants’ understanding of the questions and motivation to answer them accurately. For example, the scores for the intended future use questions, which were derived from the Consumer Information Rating Form and originally applied to paper-based health information [13], were lower than expected; some of the participants may have found it difficult to imagine using or keeping the given message they saw on the screen, even though the wording of the items was checked for understandability prior to the survey. Using the Internet guaranteeing anonymity could be expected to elicit more truthful responses, through minimizing pressure in terms of social desirability [32]. However, it is nearly impossible to eliminate the risk of information bias completely. Second, the study participants were selected from a nationwide panel provided by a research company. According to a national census [33], the percentage of the Japanese population between the ages of 35 and 44 years with a university degrees was 22.0% in 2010; this number is considerably lower than in the present study (45.6%). Therefore, the results may have been influenced to some extent by a selection bias. Third, the ages of the study participants were limited to 35–45 years. The Comprehensive Survey of Living Conditions conducted by the Japanese Ministry of Health, Labour, and Welfare revealed that individuals feeling stressed or distressed are most frequently observed in the 40–49-year-old age group (58.7% in men and 48.6% in women) [34]. In addition, the World Mental Health Japan Survey revealed that the 12-month prevalence of mental disorders was significantly higher in the younger age groups [35]. Therefore, people aged 35–45 years seemed to be suitable target for persuasive messages encouraging professional help seeking for depression. The results of the present study supports the effectiveness of depression help-seeking messages; however, it is uncertain whether the messages will work equally well in other age groups. Fourth, this study used a pretest-posttest design to assess changes in help-seeking intention for depression. In order to prevent demand characteristics, participants asked to answer a series of questions impertinent to depression between the pre and post assessments. This distractor task may have reduced the occurrence of demand characteristics to some extent; however, the effect of messaging on help-seeking intention for depression shown in this study is likely to be overestimated. Fifth, this study could not determine the causal relationships due to the cross-sectional design. Intention is recognized as a key predictor of behavior, but the strength of the intention-behavior relationship can vary depending on the type of behavior [36]. There was no knowing whether the self-reported help-seeking intentions accurately reflect the actual help-seeking behaviors if they become mentally ill. Further studies are needed to determine whether the persuasive messages developed in this study can increase actual help-seeking behaviors.

## Conclusion

The exposure to persuasive messages may promote help-seeking intentions for depression. It seems likely that loss framing will work better than neutral and gain framing. Meanwhile, the responses to persuasive messages may differ to some extent between distressed and non-distressed individuals, as individuals with psychological distress are likely to be more susceptible to persuasive messages than those without.

## Additional file

Additional file 1: Persuasive messages encouraging help-seeking for depression. (DOC 280 kb)

## Abbreviations

K6: 6-item Kessler Psychological Distress Scale
SatMDT: Step approach to Message Design and Testing
