# Supplementary material for: Responses to persuasive messages encouraging professional help seeking for depression: comparison between individuals with and without psychological distress
Source: Environ Health Prev Med. 2019 May 8;24:29. doi: 10.1186/s12199-019-0786-8 (PMC6507167; doi:10.1186/s12199-019-0786-8)
Supplement: Supplementary file 1 — Persuasive messages encouraging help-seeking for depression. (DOC 280 kb) [file 12199_2019_786_MOESM1_ESM.doc]

Additional file 1: Persuasive messages encouraging help-seeking for depression

Message 1 (neutral-framed message) – “Depression can happen to anyone”


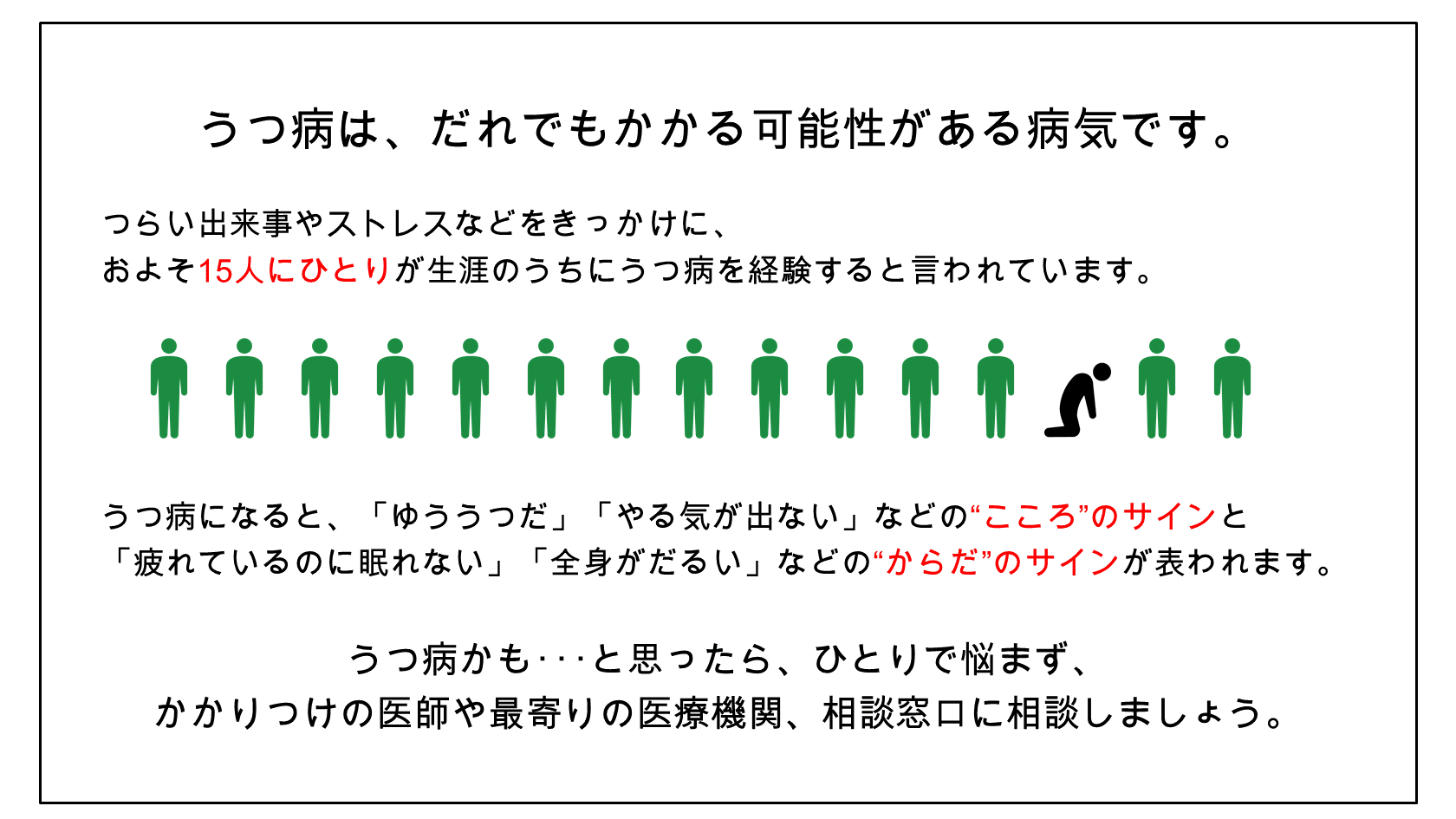


Primary message

- Depression happens to one out of 15 people.

Information on early signs of depression #1

Call to action #2

Message 2 (loss-framed message) – “Depression needs treatment”


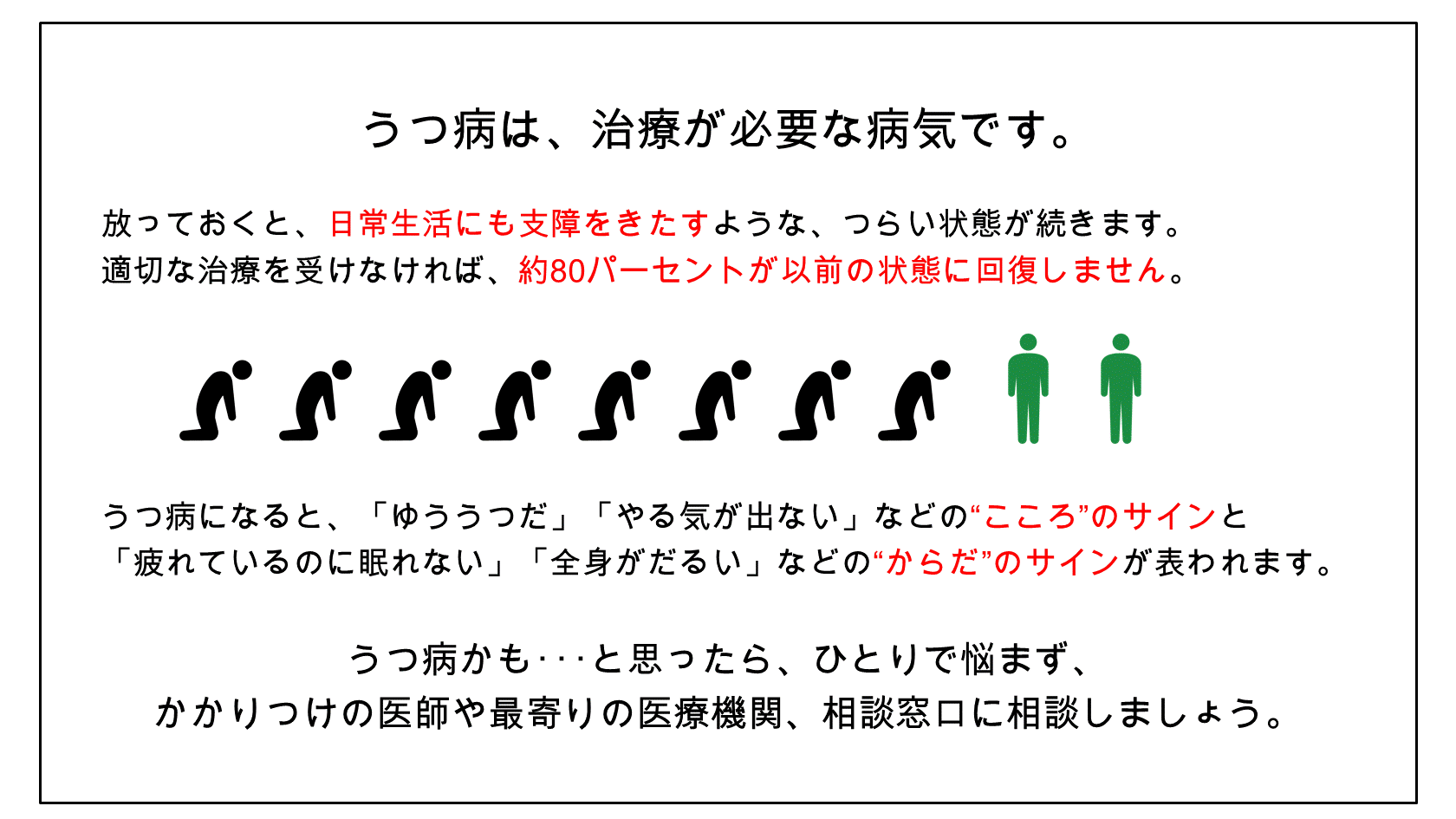


Primary message

- If not treated, 80% cannot recover from depression.

Information on early signs of depression #1

Call to action #2

Message 3 (gain-framed message) – “Depression improves with treatment”


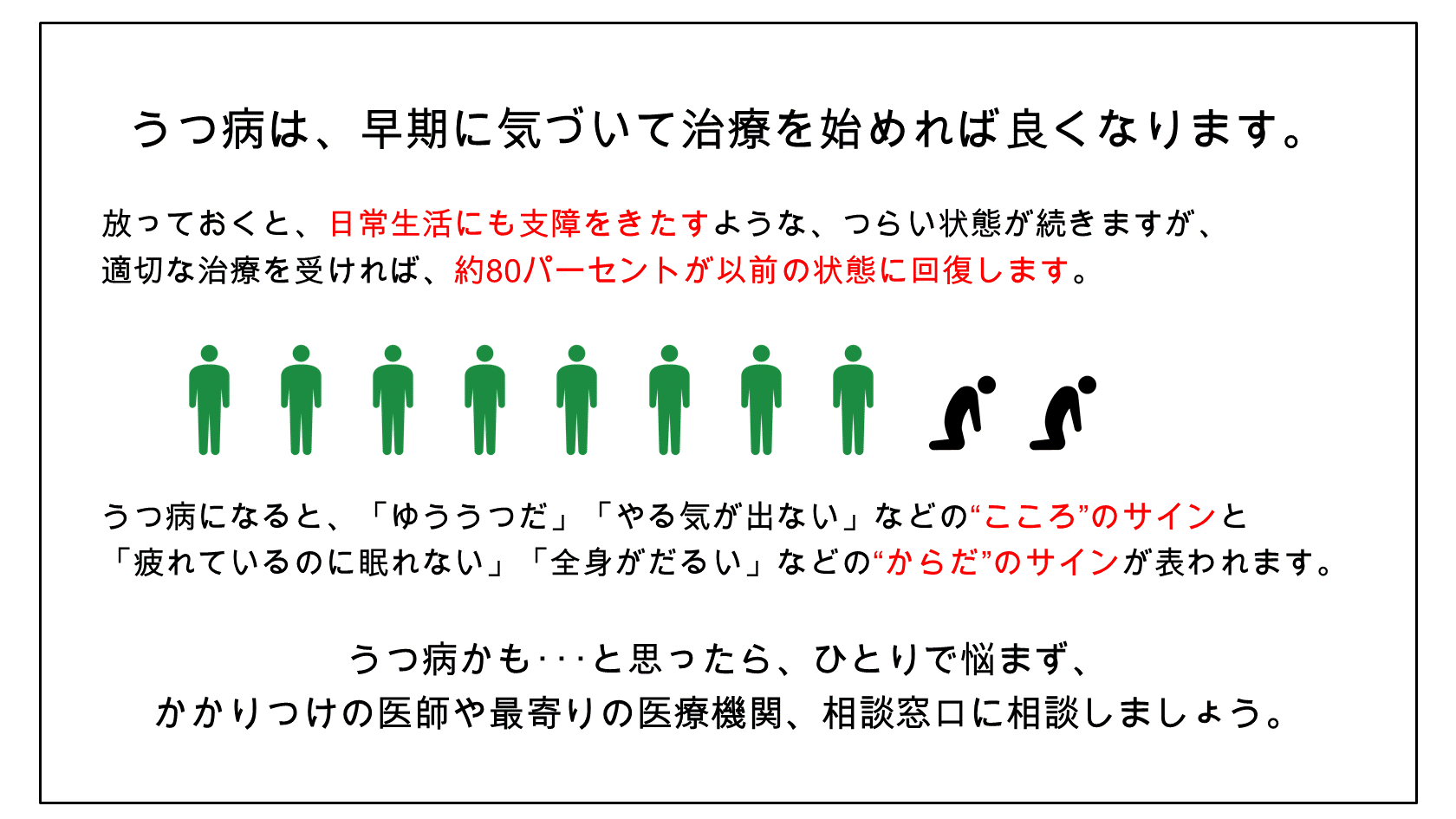


Primary message

- If treated, 80% can recover from depression.

Information on early signs of depression #1

Call to action #2

#1 Information on early signs of depression was “Depression can be recognized early by mental symptoms such as depressed mood, loss of interest, etc. and physical symptoms such as disturbed sleep, increased fatigue, etc.”

#2 Call to action was “if you think you might be depressed, don't worry alone and speak with your familiar primary care doctor.”
